# Consumption of sugar-sweetened beverages, non-sugar sweetened beverages, and their substitution and risk of type 2 diabetes: the HELIUS study

**DOI:** 10.1007/s00394-026-04043-2

**Published:** 2026-07-03

**Authors:** Sara Beigrezaei, Juliana Alexandra Hernández Vargas, Hamidreza Raeisi-Dehkordi, Lara Anne Bridge, Angeline Chatelan, Taulant Muka, Henrike Galenkamp, Barbora De Courten, Bert-Jan H. van den Born, Karien Stronks, Amin Salehi-Abargouei, Mary Nicolaou, Oscar H. Franco

**Affiliations:** 1 Department of Global Public Health and Bioethics, Julius Center for Health Sciences and Primary Care, University Medical Center (UMC) Utrecht, Utrecht, Netherlands; 2 Department of Nutrition and Dietetics, Geneva School of Health Sciences, HES-SO University of Applied Sciences and Arts Western Switzerland, Geneva, Switzerland; 3 Epistudia, Bern, Switzerland; 4 Department of Public and Occupational Health, Amsterdam UMC, Amsterdam Public Health Research Institute, University of Amsterdam, Meibergdreef 9, 1105 AZ Amsterdam Zuidoost, Netherlands; 5 Health Behaviours and Chronic Diseases, Amsterdam Public Health Amsterdam, Meibergdreef 9, 1105 AZ Amsterdam Zuidoost, Netherlands; 6 Department of Medicine, School of Clinical Sciences, Monash University, Melbourne, Australia; 7 School of Health and Biomedical Sciences, RMIT University, Bundoora, Australia; 8 Department of Internal Medicine, Section of Vascular Medicine, Amsterdam Cardiovascular Sciences, Amsterdam UMC, University of Amsterdam, Meibergdreef 9, 1105 AZ Amsterdam Zuidoost, Netherlands; 9 Nutrition Research Center, Shiraz University of Medical Sciences, Shiraz, Iran; 10 School of Nutrition and Food Sciences، Shiraz University of Medical Sciences, Shiraz, Iran; 11 Research Center for Food Hygiene and Safety, School of Public Health, Shahid Sadoughi University of Medical Sciences,, Yazd, Iran; 12 Department of Nutrition, School of Public Health, Shahid Sadoughi University of Medical Sciences, Yazd, Iran

**Keywords:** Sugar-sweetened beverages, Artificially sweetened beverages, Type 2 diabetes, Substitution analysis, Replacement, Cohort studies

## Abstract

**Purpose:**

The association between non-sugar-sweetened beverages (NSSB), as a suitable alternative for sugar-sweetened beverages (SSB), and the risk of type 2 diabetes (T2D) remains unclear. This study aimed to investigate the association between the intake of SSB and NSSB, their substitutions with each other, and T2D risk.

**Methods:**

We used a sub-sample of adults aged 18–70 years from the Healthy Life in an Urban Setting (HELIUS) study. Dietary data was assessed using ethnic-specific food frequency questionnaires. Multivariable-adjusted incidence rate ratios (IRRs) and 95% confidence intervals (CIs) for T2D risk for SSB and NSSB and their substitutions of one serving (250 ml)/day were estimated using Poisson models.

**Results:**

Among the 2612 participants included, 141 incident cases of T2D were identified. After multivariable adjustment, higher NSSB intake was associated with an increased risk of T2D in the overall population (IRR_high vs low_: 2.26; 95% CI: 1.14, 4.46) and among participants with non-Dutch ethnicity (IRR_high vs low_: 3.04; 95% CI: 1.59, 5.81). No overall association was observed between SSB intake and T2D risk, although a positive association was found among Dutch participants. In substitution analyses, replacing one serving/day of SSB with NSSB was associated with a higher T2D risk among younger participants (IRR_model 3_: 1.67; 95% CI: 1.01, 2.75), although this association was not observed in the overall population.

**Conclusion:**

These findings suggest that replacing SSBs with NSSBs may not confer a metabolic benefit and may be associated with a higher risk of T2D in specific subgroups. Further research is needed to clarify the mechanisms and subgroup-specific factors underlying these associations.

**Supplementary Information:**

The online version contains supplementary material available at 10.1007/s00394-026-04043-2.

## Introduction

Type 2 diabetes (T2D) is a leading cause of morbidity and reduced life expectancy, imposing substantial global economic and societal burdens while reaching epidemic levels [1, 2]. A wide range of factors contribute to the aetiology of T2D, including genetic, environmental, and dietary factors [3, 4]. Several dietary components have been shown to play an important role in T2D, both directly and through their association with weight gain [5].

Beverage consumption is a key component of diet composition [6]. Among them, sugar-sweetened beverages (SSB) are the main source of added sugar [7] and have been predominantly linked to an increased risk of T2D [8]. While some evidence suggests that the consumption of non-sugar-sweetened beverages (NSSB) may provide advantages for reducing T2D risk compared to SSB [9–11], several prospective studies, though not all, previously indicated that NSSB consumption may increase the risk of T2D [12–15]. Recently, NSSB have been also associated with a higher risk of insulin resistance, metabolic disorders, and T2D, raising concerns about their long-term effects on health [14–17]. Consequently, health authorities have issued conflicting recommendations regarding the substitution of NSSB for SSB [18]. Few substitution studies have quantified the risk of T2D associated with the replacement of NSSB for SSB [13, 19, 20]. A UK population-based cohort study found that substituting NSSB for SSB was associated with a 22% lower T2D incidence [21]. In contrast, findings from three large prospective cohort studies showed that substituting SSB with NSSB was not associated with T2D risk [13].

While public health authorities suggest reducing SSB intake, there is insufficient evidence to support or advise against the recommendations for alternatives, such as NSSB. Furthermore, beverage consumption patterns and the effects of dietary interventions can differ significantly across ethnic groups due to cultural preferences, genetic predispositions, and varying health perceptions [22, 23]. The present study used data from the HELIUS (Healthy Life in an Urban Setting) cohort, which includes a diverse, multi-ethnic population, offering a unique opportunity to explore these associations across different ethnic backgrounds [24]. The mixed ethnic composition of the HELIUS cohort implies a variation in dietary habits, particularly in beverage consumption. Therefore, we aimed to assess the association of SSB and NSSB intake and the effect of their substitution on the risk of T2D in a multi-ethnic population using the HELIUS cohort.

## Materials and methods

This study was conducted using the HELIUS study, a population-based prospective cohort study including adults (aged 18–70 years at inclusion) living in Amsterdam [24]. Participants were randomly sampled from the municipal registry, stratified by ethnic origin (Dutch, Surinamese, Turkish, Moroccan, and Ghanaian) [24]. More details on the study protocol are provided elsewhere [24, 25]. We used baseline data, collected between 2011 and 2015, and follow-up data, collected between 2019 and 2022. We included participants who at baseline completed an ethnic-specific food frequency questionnaire (FFQ) as part of the HELIUS Dietary Patterns study [26, 27]. For the current study, we did not include Ghanaian participants, as dietary intake in this group was measured using an FFQ with a different structure [28]. Therefore, we included Dutch, Surinamese, Turkish, and Moroccan participants with complete dietary data from the FFQ.

Of all 24,780 HELIUS participants at baseline, 5084 filled in an FFQ at baseline. Of those, 3074 were included in the first follow-up round. Furthermore, participants with the following criteria were excluded from the current study: participants diagnosed with diabetes at baseline (n = 280), participants with extreme energy intakes (< 800 kcal/day and > 4000 kcal/day for men, < 500 kcal/day and > 3500 kcal/day for women) [29] (n = 167), and participants with missing information on T2D status at follow-up (n = 15). This resulted in a study sample of 2612 participants (972 of Dutch, 549 of South-Asian Surinamese, 548 of African Surinamese, 223 of Turkish, and 320 of Moroccan origin).

The HELIUS study was approved by the Amsterdam UMC Ethics Review Board (Protocol ID NL32251.018.10, approval number 10/100# 10.17.1729) and written informed consent was obtained from all participants.

### Dietary intake assessment

Dietary intakes were collected using ethnic-specific FFQs, for Dutch, Surinamese, Turkish, and Moroccan ethnic groups, which were specially developed for the HELIUS study using an existing Dutch FFQ [27]. Each FFQ included questions on the frequency and portion size of ∼ 200 food items consumed during the past month [27]. For each FFQ, a nutrient database was constructed based on the Dutch Food Composition Table 2014 [30]. Data on ethnic-specific foods were derived from food packaging information, international food composition data, recipe calculations, and chemical analysis conducted by the RIVM–National Institute for Public Health and the Environment, where multiple samples of each food were mixed for analysis [27]. These samples were selected based on brand and different sale locations [27]. A single FFQ was used for the South-Asian Surinamese and African Surinamese groups due to similarities in commonly consumed foods [31]. For the current study, data on beverage intake included water, fruit juices, coffee/tea, SSB, and NSSB. The SSB and NSSB categories were derived from FFQ items on syrup-based drinks, soft drinks, iced teas, and energy/sport drinks, which were further distinguished according to whether they contained sugar or non-nutritive sweeteners.

### Assessment of diabetes

Diabetes was assessed both at baseline and at follow-up. Fasting blood samples were drawn during the physical examination. Glucose concentration was determined by spectrophotometry using plasma samples. Participants were asked to bring their prescribed medications to the examination, which were coded according to the Anatomical Therapeutic Chemical (ATC) classification [32]. Incident T2D was defined if the participant met at least one of the following conditions: 1) diagnosis of T2D by a health care professional according to self-report data, 2) use of T2D medications (ATC-codes A10) or 3) a fasting glucose level of ≥ 7.0 mmol/L [32]. Incident T2D was defined as not meeting one of these conditions at baseline, and meeting at least one at follow-up.

### Covariates assessment

Ethnicity was determined using data from the municipality of Amsterdam, which included the country of birth for the citizens and their parents, allowing sampling based on this indicator of birth origin [24]. Participants were considered as non-Dutch ethnicity if they were born outside of the Netherlands with at least one parent born outside of the Netherlands (first generation) or born in the Netherlands with both parents born outside the Netherlands (second generation). Thus, participants of the current study were classified as Dutch, Surinamese, Turkish, or Moroccan. Participants of Surinamese ethnic origin were further classified according to self-reported ethnic origin (obtained by questionnaire) into African, South-Asian, or other. For the Dutch sample, participants who were born in the Netherlands and whose parents were born in the Netherlands were invited. Data for other covariates including smoking status (current smoker, former smoker, never smoker), marital status (married, living together, single, divorced/separated, widow), educational level (none or elementary, lower secondary, higher secondary, tertiary education), physical activity status [moderate to high (yes, no)], and family history of diabetes (yes, no), were collected at baseline based on self-reporting. Habitual physical activity was measured using the Short Questionnaire to Assess Health (SQUASH) [33], with data converted into minutes per week spent in light and moderate to high-intensity activities, based on age-specific metabolic equivalent tasks (METs) derived from Ainsworth’s compendium of physical activity [34]. Body mass index (BMI) and body fat percentage were assessed based on the physical examination. More details on body weight and body composition measurements are provided elsewhere [24]. Energy (kcal/day) and alcohol intake (g/day) were derived from the FFQs. Hypertension was defined following the guidelines as an elevated systolic blood pressure (SBP) > 140 mmHg or diastolic blood pressure (DBP) > 90 mmHg or self-reported use of BP-lowering medication [35]. Based on the NCEP-ATP-III model guideline, hypercholesterolemia was defined as total cholesterol ≥ 5.17 mmol/L or reported lipid-lowering medication. Low high-density cholesterol (HDL) cut-offs were sex-specific as follows: men:1.034 mmol/L and women: < 1.293 mmol/L [36].

### Statistical analysis

Based on their distribution, continuous variables are presented as mean ± standard deviation (SD) or median and interquartile range (IQR), while categorical data as numbers and percentages. Differences in baseline characteristics of participants with and without T2D incidence were assessed using an independent-samples t-test or a Mann–Whitney U-test for continuous variables and a chi-squared test for categorical variables. Beverage intakes were categorized as 0 serving/day, 0–1 serving/day, and more than 1 serving/day.

We used a leave-one-out model to assess the effect of substituting SSB for NSSB and vice versa [37]. For this, one serving (250 ml/day) of SSB was replaced by the same quantity of NSSB and vice versa, while keeping the total beverage (other beverages, including natural fruit juices, coffee/tea (without sugar), and water) intake constant [38]. To examine the associations between the intake of SSB and NSSB and T2D risk, we used Poisson regression models with robust standard errors (the Huber-White sandwich estimator) to estimate incidence rate ratios (IRRs) and 95% confidence intervals (CIs). This modified Poisson approach was implemented to relax the strict equidispersion assumption of standard Poisson models and to provide valid, consistent inference in the presence of potential underdispersion or heteroscedasticity within the cohort data [39]. In all models, the natural logarithm of person-years was included as an offset term to account for varying lengths of follow-up among participants, effectively transforming the count outcome into an incidence rate [40–42]. We fitted hierarchical models using a complete case analysis as follows: crude model (adjusted for energy intake), model 1 (additionally adjusted for age, sex, and ethnicity), model 2 (additionally adjusted for smoking status, educational level, physical activity, alcohol intake, family history of diabetes, and fat percentage), and model 3 (additionally adjusted for hypertension, hypercholesterolemia, and low HDL cholesterol). The reference category for both SSB and NSSB consumption was 0 serving/day (first category). Stratified models by sex, age (18–49 y vs ≥ 50 y), BMI (< 25 kg/m^2^ vs ≥ 25 kg/m^2^) and ethnicity (Dutch vs other ethnicities (non-Dutch)) were used to evaluate different effects of these variables on the association of interest. We also tested for interaction by including a multiplicative term between the SSB and NSSB consumption categories and each of the above-mentioned potential interaction variables (sex, age, BMI, and ethnicity) in the fully adjusted model. All analyses were conducted using R version 4.4.0 (R Core Team, 2024). P values less than 0.05 were considered statistically significant.

## Results

### Baseline characteristics

The baseline characteristics of the study population are provided in Table 1 and Supplementary Fig. 1. In total, 2612 participants (1564 women and 1048 men) were included in the analysis. During a median follow-up of 7.08 years (IQR: 6.33–7.75), participants contributed 18,595.3 person-years of observation. A total of 141 incident T2D cases (5.40%) occurred, corresponding to an incidence rate of 7.58 new cases per 1000 person-years. Participants who developed T2D were older, married, South African Surinamese, with lower education, less physically active, and with a family history of diabetes. This group also showed significantly higher blood pressure, BMI, fat percentage, alcohol intake, and a higher prevalence of hypertension, low HDL, and hypercholesterolemia compared to those without T2D.

**Table 1:** Baseline characteristics of participants by T2D incidence

Variables^1,2^	All participants n = 2612	Participants without T2D at follow-up n = 2471	Participants with T2D at follow-up n = 141
Age at baseline, years	49.0 (39.0, 56.0)	48.0 (39.0, 56.0)	52.0 (47.0, 59.0)
Sex			
Female	1564 (59.9)	1489 (60.3)	75 (53.2)
Male	1048 (40.1)	982 (39.7)	66 (46.8)
Ethnicity			
Dutch	972 (37.2)	945 (38.2)	27 (19.1)
South-Asian Surinamese	549 (21.0)	500 (20.2)	49 (34.8)
African Surinamese	548 (21.0)	517 (20.9)	31 (22.0)
Turkish	223 (8.5)	211 (8.5)	12 (8.5)
Moroccan	320 (12.3)	298 (12.1)	22 (15.6)
Marital status			
Married	1121 (43.0)	1040 (42.2)	81 (57.9)
Living together	306 (11.8)	301 (12.2)	5 (3.6)
Single	780 (30.0)	753 (30.6)	27 (19.3)
Divorced or separated	352 (13.5)	331 (13.4)	21 (15.0)
Widow/widower	45 (1.7)	39 (1.6)	6 (4.3)
Educational level			
None or elementary	196 (7.5)	177 (7.2)	19 (13.6)
Lower secondary	582 (22.4)	530 (21.5)	52 (37.1)
Higher secondary	744 (28.6)	702 (28.5)	42 (30.0)
Tertiary education	1078 (41.5)	1051 (42.7)	27 (19.3)
Smoking status			
Current smoker	529 (20.3)	498 (20.2)	31 (22.1)
Former smoker	731 (28.0)	693 (28.1)	38 (27.1)
Never smoker	1348 (51.7)	1277 (51.7)	71 (50.7)
Physical activity (moderate to high)^3^			
Yes	1602 (61.3)	1526 (61.8)	76 (53.9)
No	1010 (38.7)	945 (38.2)	65 (46.1)
Family history of diabetes			
Yes	943 (40.9)	860 (39.4)	83 (65.4)
No	1365 (59.1)	1321(60.6)	44 (34.6)
Hypertension			
Yes	804 (30.8)	725 (29.4)	79 (56.0)
No	1805 (69.2)	1743 (70.6)	62 (44.0)
Hypercholesterolemia			
Yes	1315 (50.3)	1227 (49.7)	88 (62.4)
No	1297 (49.7)	1244 (50.3)	53 (37.6)
Low HDL			
Yes	540 (20.7)	483 (19.5)	57 (40.4)
No	2072 (79.3)	1988 (80.5)	84 (59.6)
SBP, mmHg	125.0 (115.5, 136.5)	124.5 (115.0, 136.1)	133.0 (123.5, 144.0)
DBP, mmHg	78.0 (71.5, 85.0)	77.5 (71.5, 85.0)	84.0 (77.5, 90.5)
BMI, kg/m^2^	25.4 (22.9, 28.4)	25.2 (22.8, 28.2)	28.7 (26.2, 33.1)
Fat percentage, %	31.3 (25.4, 37.1)	31.1 (25.4, 36.9)	33.6 (28.9, 40.5)
Energy intake, kcal/day	2061 (1644, 2538)	2061 (1646, 2538)	2076 (1612, 2513)
Alcohol intake, g/day	0.8 (0.0, 10.4)	0.8 (0.0, 10.8)	0.0 (0.0, 5.9)
SSB intake, serving^4^/day	0.1 (0.0, 0.6)	0.1 (0.0, 0.6)	0.1 (0.0, 0.6)
NSSB intake, serving/day	0.0 (0.0, 0.0)	0.0 (0.0, 0.0)	0.0 (0.0, 0.1)
Fruit juice intake, serving/day	0.1 (0.0, 0.4)	0.1 (0.0, 0.4)	0.1 (0.0, 0.6)
Water intake, serving/day	1.9 (1.0, 3.4)	1.9 (1.0, 3.4)	2.0 (1.2, 3.4)
Coffee/tea intake, serving/day	2.3 (1.3, 3.6)	2.3 (1.3, 3.6)	2.2 (1.3, 3.5)

Abbreviations: T2D, type 2 diabetes; SD, standard deviation; IQR, interquartile range; mmHg, millimeters of mercury; HDL, high-density lipoprotein; SBP, systolic blood pressure; DBP, diastolic blood pressure; BMI, body mass index; g, gram; kcal, kilocalories; SSB, sugar-sweetened beverages; NSSB, non-sugar sweetened beverages

^1^Data are presented as mean ± SD for quantitative variables with a normal distribution and as median (IQR) in case of non-normal distribution. Categorical variables are informed as absolute numbers (%)

^2^Missing values were minimal for marital status, educational level, smoking status, SBP, DBP, and BMI (< 1%), while fat percentage had 2% missingness, and family history of diabetes had the highest at 11.6%. Percentages were calculated based on the total number of participants with available data for each variable

^3^Achieving the goal of 30 min of moderate to high physical activity for five or more days

^4^ One serving = 250 ml

### SSB and NSSB intake and T2D risk

Results of the association between energy-adjusted intake of SSB and NSSB and T2D risk are presented in Table 2. Overall, higher intake of NSSB was associated with an increased risk of T2D in all multivariable-adjusted models (IRR _high vs low, model 3_: 2.26; 95% CI: 1.14, 4.46), showing also a positive trend across consumption categories (p-trend _model 3_: 0.019). Stratified analyses showed consistent associations, with higher NSSB consumption linked to higher T2D risk. Participants aged 18–49 years consuming 0–1 serving/day of NSSB had a higher risk of T2D than non-consumers (IRR _second category vs low, model 3_: 2.25; 95% CI: 1.21, 4.17). Overweight or obese subjects with high NSSB intake also had a higher incidence of T2D (IRR _high vs low, model 2_: 2.19; 95% CI: 1.06, 4.51). Higher NSSB consumption was significantly associated with increased T2D risk in non-Dutch participants (IRR _high vs low, model 3_: 3.04; 95% CI: 1.59, 5.81). A significant positive trend was found in people aged 18–49 years (all models), with overweight/obesity (models 1, 2, and 3) and Dutch origin (only model 1) and women (only model 2).

**Table 2:** The association between energy-adjusted intake of SSB and NSSB and T2D risk

	SSB serving category				NSSB serving category			
	0	0–1	> 1	*p*-trend	0	0–1	> 1	*p*-trend
	IRR (95% CI)	IRR (95% CI)	IRR (95% CI)		IRR (95% CI)	IRR (95% CI)	IRR (95% CI)	
All participants								
No. of participants/cases	778/41	1436/77	398/23		1825/92	682/39	105/10	
Crude	1 (ref.)	1.01 (0.70, 1.46)	1.06 (0.64, 1.75)	0.860	1 (ref.)	1.14 (0.79, 1.63)	1.81 (0.97, 3.37)	0.117
Model 1	1 (ref.)	1.19 (0.83, 1.71)	1.45 (0.88, 2.40)	0.140	1 (ref.)	1.22 (0.85, 1.75)	2.39 (1.29, 4.42)	0.021
Model 2	1 (ref.)	0.96 (0.67, 1.39)	1.07 (0.59, 1.93)	0.935	1 (ref.)	1.29 (0.89, 1.88)	2.47 (1.30, 4.69)	0.013
Model 3	1 (ref.)	1.01 (0.70, 1.45)	1.03 (0.57, 1.87)	0.938	1 (ref.)	1.31 (0.90, 1.91)	2.26 (1.14, 4.46)	0.019
Based on sex								
Men	273/17	575/36	200/13		763/45	245/16	40/5	
Crude	1 (ref.)	1.01 (0.58, 1.75)	1.09 (0.54, 2.22)	0.839	1 (ref.)	1.12 (0.64, 1.94)	2.06 (0.86, 4.94)	0.219
Model 1	1 (ref.)	1.14 (0.66, 1.96)	1.41 (0.70, 2.81)	0.357	1 (ref.)	1.11 (0.64, 1.93)	2.49 (1.06, 5.84)	0.151
Model 2	1 (ref.)	1.01 (0.58, 1.74)	1.09 (0.47, 2.50)	0.869	1 (ref.)	1.27 (0.72, 2.25)	2.14 (0.82, 5.60)	0.138
Model 3	1 (ref.)	0.99 (0.57, 1.73)	1.01 (0.43, 2.39)	0.998	1 (ref.)	1.25 (0.71, 2.21)	2.15 (0.76, 6.05)	0.160
Women	505/24	861/41	198/10		1,062/47	437/23	65/5	
Crude	1 (ref.)	0.99 (0.61, 1.62)	1.01 (0.49, 2.08)	0.995	1 (ref.)	1.19 (0.73, 1.93)	1.62 (0.68, 3.90)	0.262
Model 1	1 (ref.)	1.21 (0.75, 1.95)	1.50 (0.72, 3.09)	0.264	1 (ref.)	1.31 (0.81, 2.12)	2.25 (0.94, 5.38)	0.073
Model 2	1 (ref.)	0.99 (0.61, 1.61)	1.20 (0.54, 2.66)	0.788	1 (ref.)	1.36 (0.83, 2.24)	2.48 (1.00, 6.12)	0.046
Model 3	1 (ref.)	1.03 (0.63, 1.70)	1.29 (0.58, 2.85)	0.638	1 (ref.)	1.41 (0.85, 2.32)	1.89 (0.74, 4.81)	0.083
Based on age								
18–49 years	319/8	798/31	262/10		922/26	394/19	63/4	
Crude	1 (ref.)	1.48 (0.70, 3.15)	1.36 (0.54, 3.46)	0.504	1 (ref.)	1.70 (0.95, 3.03)	2.09 (0.76, 5.78)	0.035
Model 1	1 (ref.)	1.75 (0.84, 3.66)	2.04 (0.83, 5.01)	0.091	1 (ref.)	1.88 (1.05, 3.35)	2.82 (0.97, 8.19)	0.009
Model 2	1 (ref.)	1.50 (0.67, 3.38)	1.14 (0.41, 3.22)	0.751	1 (ref.)	2.27 (1.20, 4.28)	2.95 (0.89, 9.86)	0.003
Model 3	1 (ref.)	1.71 (0.74, 3.96)	1.02 (0.36, 2.89)	0.950	1 (ref.)	2.25 (1.21, 4.17)	2.92 (0.94, 9.10)	0.003
≥ 50 years	459/33	638/46	136/13		903/66	288/20	42/6	
Crude	1 (ref.)	1.01 (0.66, 1.56)	1.32 (0.71, 2.45)	0.528	1 (ref.)	0.96 (0.60, 1.55)	1.90 (0.87, 4.17)	0.416
Model 1	1 (ref.)	1.02 (0.67, 1.57)	1.31 (0.71, 2.42)	0.521	1 (ref.)	0.94 (0.58, 1.52)	2.30 (1.08, 4.88)	0.347
Model 2	1 (ref.)	0.81 (0.53, 1.24)	1.16 (0.56, 2.40)	0.887	1 (ref.)	0.95 (0.58, 1.55)	2.15 (0.98, 4.73)	0.332
Model 3	1 (ref.)	0.83 (0.54, 1.26)	1.16 (0.56, 2.43)	0.932	1 (ref.)	0.97 (0.60, 1.58)	1.98 (0.85, 4.61)	0.364
Based on BMI								
< 25 kg/m^2^	338/4	664/15	196/5		861/18	296/5	41/1	
Crude	1 (ref.)	1.97 (0.67, 5.86)	2.31 (0.60, 8.94)	0.178	1 (ref.)	0.81 (0.31, 2.15)	1.13 (0.16, 8.24)	0.814
Model 1	1 (ref.)	2.50 (0.85, 7.37)	3.50 (0.89, 13.78)	0.043	1 (ref.)	1.01 (0.39, 2.68)	1.55 (0.22, 11.23)	0.797
Model 2	1 (ref.)	1.81 (0.57, 5.75)	1.91 (0.36, 10.33)	0.391	1 (ref.)	1.11 (0.45, 2.73)	2.04 (0.29, 14.56)	0.581
Model 3	1 (ref.)	2.19 (0.63, 7.61)	1.79 (0.35, 9.38)	0.385	1 (ref.)	1.06 (0.43, 2.62)	2.00 (0.23, 17.33)	0.640
≥ 25 kg/m^2^	439/37	771/62	202/18		963/74	385/34	64/9	
Crude	1 (ref.)	0.93 (0.63, 1.36)	0.95 (0.55, 1.66)	0.773	1 (ref.)	1.15 (0.78, 1.70)	1.73 (0.91, 3.30)	0.140
Model 1	1 (ref.)	1.03 (0.71, 1.51)	1.17 (0.68, 2.04)	0.625	1 (ref.)	1.20 (0.82, 1.77)	2.18 (1.14, 4.20)	0.046
Model 2	1 (ref.)	0.88 (0.60, 1.29)	0.96 (0.51, 1.80)	0.708	1 (ref.)	1.29 (0.86, 1.94)	2.43 (1.22, 4.84)	0.021
Model 3	1 (ref.)	0.90 (0.62, 1.32)	0.94 (0.50, 1.77)	0.711	1 (ref.)	1.31 (0.88, 1.97)	2.19 (1.06, 4.51)	0.030
Based on ethnicity								
Dutch	390/12	495/10	87/5		652/15	253/8	67/4	
Crude	1 (ref.)	0.68 (0.30, 1.57)	1.98 (0.65, 6.05)	0.685	1 (ref.)	1.35 (0.58, 3.12)	2.51 (0.86, 7.34)	0.121
Model 1	1 (ref.)	0.92 (0.40, 2.13)	3.58 (1.19, 10.82)	0.215	1 (ref.)	1.63 (0.72, 3.72)	2.97 (1.00, 8.45)	0.037
Model 2	1 (ref.)	0.80 (0.33, 1.95)	3.08 (1.06, 8.97)	0.307	1 (ref.)	1.76 (0.77, 4.03)	2.52 (0.85, 7.51)	0.057
Model 3	1 (ref.)	0.78 (0.31, 2.00)	2.80 (1.02, 7.72)	0.345	1 (ref.)	1.74 (0.76, 3.98)	2.38 (0.75, 7.56)	0.083
Other ethnicities	388/29	941/67	311/18		1,173/77	429/31	38/6	
Crude	1 (ref.)	0.90 (0.59, 1.35)	0.65 (0.37, 1.17)	0.143	1 (ref.)	1.11 (0.75, 1.66)	2.30 (1.07, 4.93)	0.156
Model 1	1 (ref.)	0.94 (0.63, 1.40)	0.73 (0.41, 1.30)	0.279	1 (ref.)	1.18 (0.79, 1.76)	2.88 (1.35, 6.15)	0.060
Model 2	1 (ref.)	0.93 (0.61, 1.40)	0.70 (0.35, 1.39)	0.308	1 (ref.)	1.23 (0.81, 1.86)	3.30 (1.67, 6.49)	0.038
Model 3	1 (ref.)	1.00 (0.66, 1.52)	0.73 (0.37, 1.45)	0.409	1 (ref.)	1.25 (0.82, 1.91)	3.04 (1.59, 5.81)	0.034

Abbreviations: T2D, type 2 diabetes; SSB, sugar-sweetened beverages; NSSB, non-sugar-sweetened beverages; IRR, incidence rate ratio; CI, confidence interval; BMI, body mass index

*Other ethnicities: South-Asian Surinamese, African Surinamese, Turkish, and Moroccan

Crude: adjusted for total energy intake. The sample size for this model was 2612

Model 1: additionally adjusted for age, sex, and ethnicity. The sample size for this model was 2612

Model 2: additionally adjusted for smoking status, educational level, physical activity, alcohol intake, family history of diabetes, and fat percentage. The sample size for this model was 2243

Model 3: additionally adjusted for hypertension, hypercholesterolemia and low HDL cholesterol. The sample size for this model was 2240

No significant association was found between SSB intake and T2D risk in the overall sample or stratified models, except in the Dutch subgroup, where high vs. low consumption showed significance in all multivariable models (Table 2). We found no statistically significant interaction between SSB and NSSB consumption categories and the potential interaction variables (sex, age, BMI and ethnicity).

### Substitution of SSB and NSSB intake and T2D risk

Table 3 shows the association between substituting SSB intake for NSSB and vice versa, and T2D risk. In the overall population, replacing one serving/day of SSB with NSSB was associated with a higher risk of T2D in multivariable-adjusted models, reaching statistical significance in models 1 and 2 (IRR model 2: 1.48; 95% CI: 1.06, 2.06). Conversely, replacing NSSB with SSB was associated with a lower risk of T2D in adjusted models, but was statistically significant only in models 1 and 2 (IRR model 2: 0.68; 95% CI: 0.49, 0.94).

**Table 3:** Associations between substituting a serving/day of SSB with NSSB and vice versa and T2D risk

		Substituting of SSB with NSSB				Substituting of NSSB with SSB			
Per 1 serving/day		Crude IRR (95% CI)	Model 1 IRR (95% CI)	Model 2 IRR (95% CI)	Model 3 IRR (95% CI)	Crude IRR (95% CI)	Model 1 IRR (95% CI)	Model 2 IRR (95% CI)	Model 3 IRR (95% CI)
All participants		1.30 (0.96, 1.76)	1.35 (1.02, 1.80)	1.48 (1.06, 2.06)	1.40 (0.97, 2.02)	0.77 (0.57, 1.05)	0.74 (0.56, 0.98)	0.68 (0.49, 0.94)	0.71 (0.49, 1.03)
Based on sex	Men	1.26 (0.78, 2.04)	1.24 (0.78, 1.96)	1.27 (0.72, 2.24)	1.30 (0.70, 2.40)	0.79 (0.40, 1.29)	0.81 (0.51, 1.28)	0.79 (0.45, 1.39)	0.77 (0.42, 1.43)
	Women	1.34 (0.92, 1.94)	1.46 (1.02, 2.09)	1.64 (1.07, 2.52)	1.37 (0.87, 2.16)	0.75 (0.52, 1.08)	0.68 (0.48, 0.98)	0.61 (0.40, 0.93)	0.73 (0.46, 1.16)
Based on age	18–49 years	1.28 (0.79, 2.05)	1.28 (0.82, 1.99)	1.69 (1.04, 2.72)	1.67 (1.01, 2.75)	0.78 (0.49, 1.26)	0.78 (0.50, 1.22)	0.59 (0.37, 0.96)	0.60 (0.36, 0.99)
	≥ 50 years	1.31 (0.90, 1.92)	1.42 (0.98, 2.07)	1.34 (0.86, 2.09)	1.27 (0.78, 2.05)	0.76 (0.52, 1.11)	0.70 (0.48, 1.02)	0.74 (0.48, 1.16)	0.79 (0.49, 1.27)
Based on BMI	< 25 kg/m^2^	0.90 (0.32, 2.51)	1.07 (0.43, 2.66)	1.48 (0.55, 3.98)	1.35 (0.48, 3.77)	1.11 (0.40, 3.11)	0.94 (0.38, 2.34)	0.68 (0.25, 1.81)	0.74 (0.27, 3.08)
	≥ 25 kg/m^2^	1.27 (0.92, 1.74)	1.32 (0.96, 1.80)	1.45 (1.03, 2.05)	1.39 (0.95, 2.03)	0.79 (0.57, 1.09)	0.76 (0.56, 1.04)	0.69 (0.49, 0.97)	0.72 (0.49, 1.05)
Base on ethnicity	Dutch	1.24 (0.75, 2.07)	1.07 (0.63, 1.82)	1.10 (0.67, 1.82)	1.03 (0.59, 1.77)	0.80 (0.48, 1.34)	0.94 (0.55, 1.60)	0.91 (0.55, 1.50)	0.98 (0.57, 1.69)
	Other ethnicities*	1.73 (1.14, 2.64)	1.78 (1.20, 2.63)	2.10 (1.35, 3.26)	2.03 (1.29, 3.19)	0.58 (0.38, 0.88)	0.56 (0.38, 0.84)	0.48 (0.31, 0.74)	0.49 (0.31, 0.78)

Abbreviations: T2D, type 2 diabetes; SSB, sugar-sweetened beverages; NSSB, non-sugar-sweetened beverages; IRR, incidence rate ratio; CI, confidence interval; BMI, body mass index

^*^Other ethnicities: South-Asian Surinamese, African Surinamese, Turkish, and Moroccan

Crude: adjusted for total energy intake. The sample size for this model was 2612

Model 1: additionally adjusted for age, sex, and ethnicity. The sample size for this model was 2612

Model 2: additionally adjusted for smoking status, educational level, physical activity, alcohol intake, family history of diabetes, and fat percentage. The sample size for this model was 2243

Model 3: additionally adjusted for hypertension, hypercholesterolemia and low HDL cholesterol. The sample size for this model was 2240

Stratified analyses showed that these substitution associations were particularly evident among participants aged 18–49 years and among participants with overweight or obesity. Among younger participants, replacing SSB with NSSB was associated with increased T2D risk (IRR _model 3_: 1.67; 95% CI: 1.01, 2.75), whereas replacing NSSB with SSB was associated with lower T2D risk (IRR _model 3_: 0.60; 95% CI: 0.36, 0.99). Similarly, among participants of non-Dutch origin, substituting SSB with NSSB was associated with a higher T2D risk across all models, while substituting NSSB with SSB was associated with lower T2D risk.

## Discussion

In this prospective population-based cohort study, higher NSSB intake was consistently associated with increased T2D risk, with a significant positive trend across consumption categories. This association remained significant across subgroups, particularly among individuals with overweight or obesity and participants with a non-Dutch background. In contrast, SSB intake showed no overall association with T2D risk, although a positive association was observed among Dutch participants. In subgroup analyses, substituting SSB with NSSB was associated with a higher T2D risk among younger participants, individuals with overweight or obesity, and non-Dutch participants, whereas substituting NSSB with SSB was associated with a lower T2D risk among younger and non-Dutch participants. These findings suggest that NSSB consumption and beverage substitution patterns may reflect underlying metabolic vulnerability or health-related behavioral change, particularly in specific subgroups.

Several studies and meta-analyses have consistently shown an association between the consumption of SSB and T2D risk [43, 44]; however, in the present study, SSB intake was not associated with the incidence of T2D. Several factors may explain this null finding. First, the relatively low intake and limited variation in SSB consumption within our sample may have reduced our ability to detect an association. Second, SSB intake was assessed only once at baseline using an FFQ, which may not have fully captured habitual or changing beverage consumption over time and may have attenuated associations because of measurement error. In addition, grouping different types of SSBs together may have masked heterogeneous associations, and residual confounding by lifestyle or dietary factors cannot be excluded. Finally, the absence of an overall association may also reflect heterogeneity across ethnic groups, as a positive association between SSB intake and T2D was observed only among Dutch participants. Nonetheless, SSB consumption has consistently been identified as an important contributor to T2D risk in the broader literature. Despite public health efforts that emphasize reducing SSB intake, current evidence is conflicting regarding appropriate substitute beverages to reduce the risk of chronic diseases. In our study, NSSB intake was significantly associated with increased T2D risk, which was persistent among subjects with overweight and obesity; however, these findings should be viewed in light of the limited sample size and number of incident T2D cases in the subgroup analyses. Other prospective observational studies have also found associations between a higher NSSB intake and a higher risk of T2D. For instance, in a large prospective cohort of 105,588 French adults, total sweeteners, aspartame, sucralose, and acesulfame-K, from the overall diet, were associated with a higher risk of T2D [12]. Moreover, in the Nurses’ Health Study (NHS) and NHS II, an increase in NSSB of 0.50 serving/day or more was associated with an 18% increased T2D risk [13]. Additionally, the Women’s Health Initiative showed a higher risk of T2D for subjects with higher NSSB intake (≥ 2 servings/day) compared to those with lower intake (never or < 3 servings/month) [44]. The European Prospective Investigation into Cancer and Nutrition (EPIC)-InterAct cohort reported that a daily one-can increase of NSSB was related to an increased T2D risk; however, after adjustment for BMI, the association became nonsignificant [45]. Moreover, a meta-analysis by Azad et al. [14] showed an association between the highest versus lowest intake of NSSB and a higher risk of T2D.

Several mechanisms supporting links between the intake of NSSB and health outcomes have been proposed. Although NSSBs contain lower sugar and calorie content compared to SSB, most of the non-nutritive sweeteners in NSSB may not be biologically inert. Research suggests that they have the potential to trigger inflammatory responses, insulin resistance, appetite dysregulation, and alter gut microbiota composition and function [46, 47]. Animal studies have shown that non-nutritive sweeteners can provoke reactive oxygen species production and inflammation in the liver and brain [48, 49]. Egan and Margolskee [50] reported that healthy adults have shown individual-specific effects of non-nutritive sweeteners on the gut microbiome, leading to increased glycemic responses. Moreover, they found some specific gut microbial features associated with artificial sweetener consumption, such as increased levels of kynurenine (a metabolite related to diabetes) and an overrepresentation of T2D-related biosynthetic pathways. It has been suggested that the interaction between non-nutritive sweeteners and sweet-taste receptors in the intestines might stimulate the secretion of glucagon-like peptide 1 [50]. This could potentially decrease blood glucose levels by increasing insulin secretion, leading to an increase in appetite and subsequent weight gain [50]. Furthermore, non-nutritive sweeteners and other food additives could induce excessive secretion from pancreatic islet cells. This may lead to hepatic insulin resistance and increased fat accumulation, which are risk factors for T2D [51].

According to our findings, substituting one serving of SSB with NSSB was not associated with a lower risk of T2D in the overall population, which is in line with previous studies [13, 20]. However, in subgroup analyses, replacing SSB with NSSB was associated with a higher risk of T2D among younger participants (18–49 years), individuals with overweight or obesity, and participants with a non-Dutch background. Conversely, replacing NSSB with SSB was associated with a lower T2D risk among younger and non-Dutch participants. These findings suggest that the metabolic implications of beverage substitution may differ across population subgroups and should be interpreted with caution. Data from the EPIC-Norfolk Study showed that replacing NSSB for SSB did not significantly reduce T2D risk [52]. Moreover, the results from three large prospective U.S. cohorts showed that substituting one daily serving of SSB for NSSB was not associated with a lower risk of T2D [13]. Further research is needed to assess the effects of substituting NSSB for SSB in diverse populations. Additionally, our study adds novel insight by identifying subgroup-specific substitution patterns, particularly among younger and non-Dutch participants, which may reflect underlying behavioral, dietary, or metabolic differences and warrant further investigation.

We observed differing patterns across subgroups, particularly by ethnicity, age, and BMI. Positive associations between NSSB intake and T2D were observed among participants with a non-Dutch background, whereas a positive association for SSB intake was observed only among Dutch participants. In addition, substitution analyses showed that replacing SSB with NSSB was associated with a higher T2D risk among younger participants, individuals with overweight or obesity, and non-Dutch participants, while replacing NSSB with SSB was associated with a lower T2D risk in some of these subgroups. At first glance, these findings may appear counterintuitive. However, they likely reflect different underlying epidemiological contrasts rather than a protective effect of SSB. Specifically, NSSB consumption may be more common among individuals who are already at elevated metabolic risk and who may have changed their beverage choices in response to weight concerns, prediabetes, or perceived health risks [11]. In this context, NSSB intake and substitution toward NSSB may act as markers of underlying metabolic vulnerability or health-related behavioral change rather than direct causal determinants of T2D risk. The differing associations observed across ethnic groups may also reflect variation in beverage consumption patterns, accompanying dietary habits, sociocultural context, or health awareness. However, given the limited sample size and number of incident T2D cases in subgroup analyses, future studies with larger multi-ethnic populations are needed to establish the robustness of these findings.

This study has several limitations that should be considered. The small sample size of our study, combined with the low incidence of T2D in general and particularly in subgroup analyses, may have affected the associations between the intake of SSB, NSSB, their substitution, and the incidence of T2D. Additionally, the low frequency of NSSB consumption in our sample may have further constrained the power of our analyses, particularly when examining specific subgroups. Furthermore, dietary intake data were available only for a subset of HELIUS participants, and the present analysis was therefore restricted to those with complete FFQ data. As a result, selection bias cannot be excluded if included participants differed systematically from those not included, which may have influenced the observed associations and may limit the generalizability of the findings. Several limitations of the present study are typical for observational studies, impeding causal inference for the effect of diet on disease incidence. Measurement errors in the intake of SSB and NSSB, or other dietary components in epidemiological studies are inevitable. Additionally, it is possible that nondifferential misclassification may have biased the results towards the null hypothesis. Moreover, the variation in SSB and NSSB consumption might have been limited, which may have reduced our ability to detect associations with T2D risk. It is important to note that dietary consumption of beverages and other items was assessed only once at baseline, without considering potential modifications to dietary patterns during follow-up. Although we tried to adjust for potential confounding factors, we could not entirely rule out the possibility of unmeasured or residual confounding that might affect the observed associations. In particular, the substitution analyses may partly reflect broader dietary and behavioral contexts in which SSBs and NSSBs are consumed, rather than the isolated metabolic effect of directly replacing one beverage with the other. Because these beverages may be consumed alongside different foods, meal patterns, or eating occasions, residual confounding by accompanying dietary behaviors cannot be excluded, especially across ethnically diverse populations. In our study, the possibility of reverse causality, whereby individuals at higher risk of T2D, including those with overweight, obesity, or prediabetes, may preferentially choose NSSBs as a strategy to reduce sugar intake, manage body weight, or maintain weight loss [16], emerges as a notable concern when interpreting the results. This may lead to an overestimation of the association between NSSB consumption and T2D risk. We tried to address this limitation through subgroup analyses, in which the association between NSSB intake and T2D risk was also observed among participants with overweight or obesity. Nevertheless, the possibility of reverse causation cannot be completely excluded. In addition, it has been demonstrated that participants with higher NSSB intake often exhibit other unhealthy lifestyle behaviors that could potentially increase the risk of cardiometabolic diseases [53].

## Conclusion

Higher intake of NSSBs was associated with an increased risk of T2D, with consistent positive associations across key subgroups, particularly among individuals with overweight or obesity and participants with a non-Dutch background. In contrast, SSB intake was not associated with T2D risk in the overall population, although subgroup-specific associations were observed. Substituting SSBs with NSSBs was not linked to a lower T2D risk in the overall population and was associated with a higher T2D risk in several subgroups, including younger, overweight/obese, and non-Dutch participants. While NSSBs are often promoted as healthier alternatives to SSBs, our findings highlight the need for further interventional and mechanistic studies to better understand their long-term health effects and to inform public health recommendations.

## Supplementary Information

Below is the link to the electronic supplementary material.

Supplementary Material 1

## Data Availability

The HELIUS data are owned by the Amsterdam University Medical Centers, located at AMC in Amsterdam, The Netherlands. Any researcher can request the data by submitting a proposal to the HELIUS Executive Board as outlined at http://www.heliusstudy.nl/en/researchers/collaboration, by email: heliuscoordinator@amsterdamumc.nl. The HELIUS Executive Board will check proposals for compatibility with the general objectives, ethical approvals and informed consent forms of the HELIUS study. There are no other restrictions to obtaining the data and all data requests will be processed in the same manner.
