# Supplementary material for: Consumption of sugar-sweetened beverages, non-sugar sweetened beverages, and their substitution and risk of type 2 diabetes: the HELIUS study
Source: Eur J Nutr. 2026 Jul 3;65(5):192. doi: 10.1007/s00394-026-04043-2 (PMC13331891; doi:10.1007/s00394-026-04043-2)
Supplement: Supplementary file 1 — Supplementary Material 1 [file 394_2026_4043_MOESM1_ESM.docx]

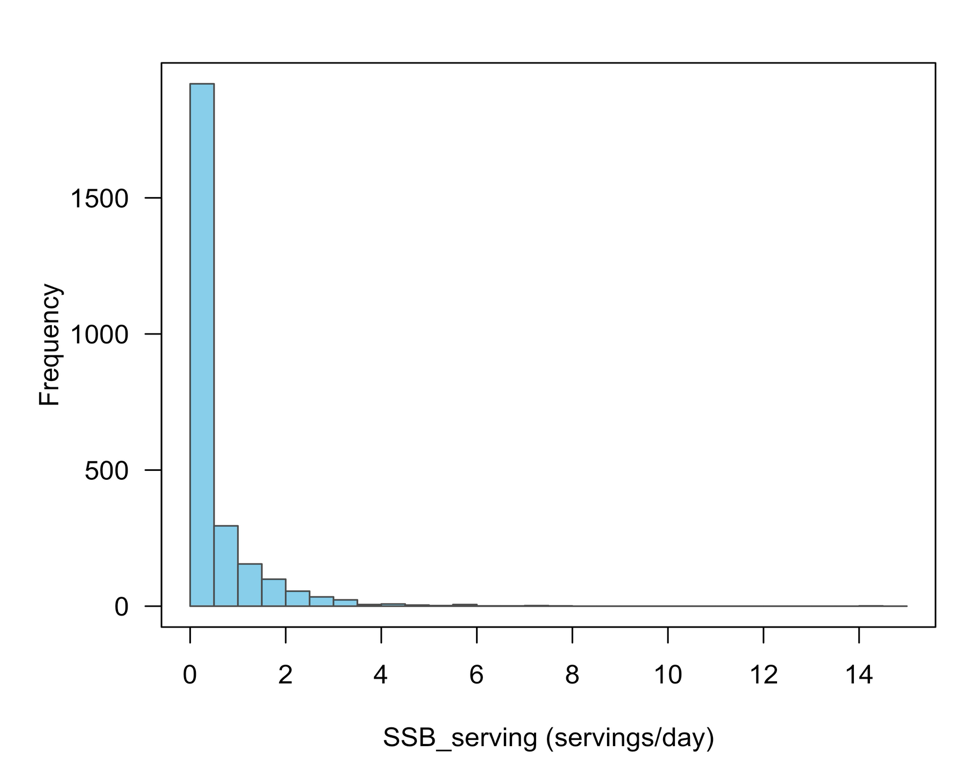

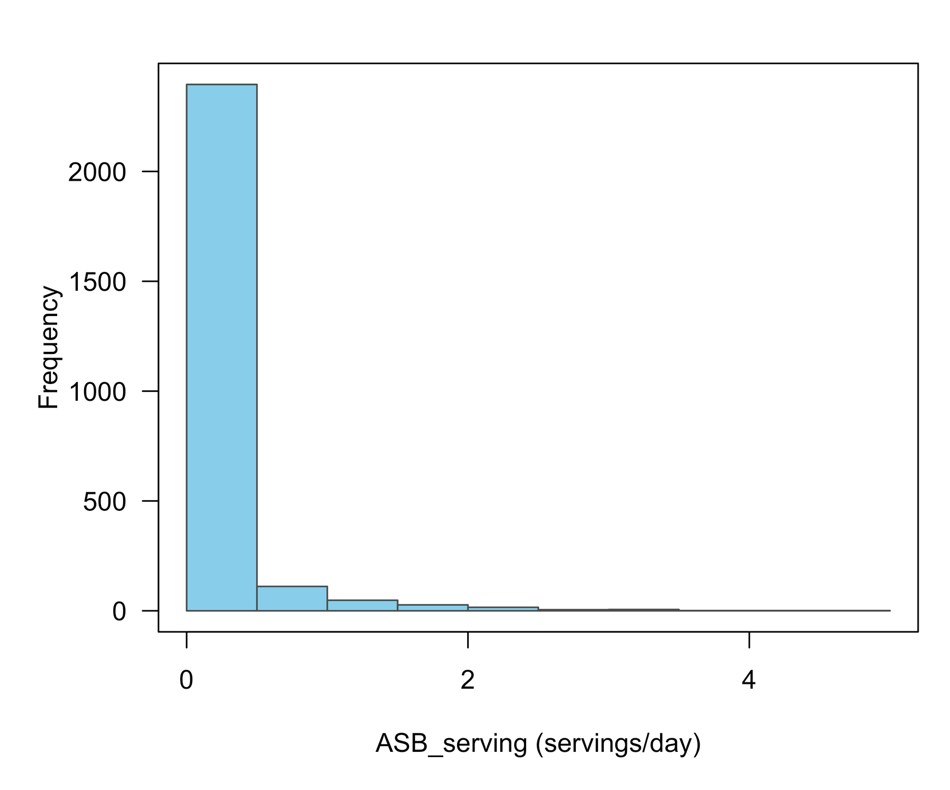


Frequency

Frequency

NSSB intake (serving/day)

SSB intake (serving/day)

Supplementary Figure 1: Distribution of NSSB and SSB intake in servings/day
